# A review on the pesticides in coffee: Usage, health effects, detection, and mitigation

**DOI:** 10.3389/fpubh.2022.1004570

**Published:** 2022-11-08

**Authors:** Areej Merhi, Rita Kordahi, Hussein F. Hassan

**Affiliations:** Department of Natural Sciences, School of Arts and Sciences, Lebanese American University, Beirut, Lebanon

**Keywords:** coffee, pesticide residues, analytical methods, mitigation, beverage

## Abstract

Coffee is considered among the most popular beverages and is classified as the second most exported item worldwide. The presence of pesticides in this staple commodity is a challenge to import and export activities, in addition to the fact that pesticides are toxins of public health concern. Even if pesticides are applied properly and their residues are within the acceptable range, it is important to know the fate of these pesticides prior to their ingestion. A plethora of research has been done to optimize methods and thus to have valid procedures to test for the presence of pesticides in coffee. In this review, the analytical methods used in these articles to detect and quantify the pesticides in coffee beans, roasted coffee, and coffee infusion were identified. This review highlights as well the main factors that play a key role in having good separation, identification, and recovery of pesticide residues in the aforementioned items. In addition, the review explains the effect of pesticides on human health and the mitigation techniques for pesticide exposure.

## Introduction

Coffee is part of the *Rubiaceae* family, which is a type of flowering plant with seeds producing coffee. It is estimated that 75–80% of the world's coffee manufacturing is from *Coffea arabica* and about 20% is from *C. canephora* (1, 2). Coffee is classified among the most consumed beverages in the world (3). Coffee beverages are produced from ground roasted beans of *Coffea spp* plant. The main commercialized coffee species related to *genus Coffea* are *Coffea arabica, Coffea canephora* and *Coffea liberica* (4, 5). Coffee is known for its enhanced impact on the economy in tropical agricultural regions due to its high rate of exportation. It has a global market and is considered, after oil, the most merchandised product in the world (6). Coffee is rich in valuable antioxidants mainly chlorogenic acid (1).

As with any crop, pests and plant diseases can affect the coffee plants. To avoid diseases that control the quality and quantity of production, pesticides are used at different stages of cultivation of coffee crops (7). The World Health Organization and the Food and Agricultural Organization have defined pesticides as: any substance or a mixture of substances intended for repelling, destroying, or controlling any pest during or interfering in the production, processing, storage, or marketing of food (8). An ideal pesticide should be very selective, effective for pest control for a determined period of time and degrading afterwards (9). Insecticides are mainly used in coffee crops. Insecticides include organophosphates (OP), pyrethroids, and carbamates.

After spraying the plants with pesticides, the residues might be present in the crop which might lead to harmful health conditions in humans due to their toxicity. However, time-temperature steps determine the chemical composition and characteristics of coffee such as flavor, aroma, and color (10). Prior to roasting the green coffee beans, they are dried at a temperature < 160°C. Then, roasting takes place by increasing the temperature up to 260°C. Important chemical reactions take place when the temperature reaches 190°C such as pyrolytic reactions including hydrolysis, decarboxylation, oxidation, and reduction. Even after these steps, studies have shown that pesticides have the capacity to still exist in commercial coffee. This makes pesticide residues in coffee a major health concern and monitoring the pesticides is crucial since unintended exposure to pesticides can occur *via* consuming coffee (1).

In addition to health problems, environmental contamination could be another consequence of using pesticides. To answer the question about the fate of pesticides after being sprayed, an attempt to evaluate and predict their destination was presented using the level I fugacity model (11). The modeling studies (Fugacity Level I) confirm that pesticides are mostly abundant in the sediment when it comes to concentration in the environment but without considering the volume of the compartments. Regarding the percentage and the volume of the compartments, pesticides were mostly abundant in the soil. Therefore, sediment and soil had the highest risk when it comes to the evaluation of pesticides. However, more attempts are in progress to use other levels of modeling (fugacity), while taking into consideration environmental characteristics where advection, degradation, emission, and transfer of substances between compartments occur. We recommend being selective when it comes to choosing the type and quantity of the pesticides used and to continue monitoring their fate afterwards (11).

Since coffee, along with other agricultural waste, contains important nutrients, the defective coffee bean can be a solution to the problem. A study showed the function of micro niches in the porous surface of the green coffee bean in the beans' bacterial biodegradation of dichlorodiphenyltrichloroethane (DDT) and endosulfan (12). Scanning Electron Microscope (SEM) analysis was conducted to evaluate the changes occurring on the surface of the coffee beans, when cultivating them. The coffee beans were air dried, ground, and sieved. Afterwards, the isolation of organochlorine pesticide degrading bacteria from coffee beans was performed, followed by the bacterial inoculum preparation. However, coffee beans contain important amounts of carbon and nutrients which promote bacterial growth (13). Anaerobic and aerobic conditions promote electrophilic aromatic contaminants (such as organochlorine and azo) mineralization (14). In other words, defective green coffee beans contain important nutrients that could be used in organochlorine pesticide degrading bacteria in liquid media. It is worth mentioning that DDT is an organochlorine pesticide that was banned in agriculture. Organochlorine pesticides have halogen electron removing groups which create electron deficiency in the molecule leading to the resistance of aerobic degradation (15). When reductive states occur, those pesticides will be more prone to attack especially when introducing auxiliary electron donors. Therefore, DDT mineralization needs a combination of environmental states (redox potential, pH, co-substrate, pollutant concentration, etc.). Currently, agro-industrial wastes demonstrated positive effects in the toxic organopollutant biodegradation improvement (16–18).

In short, analyzing coffee samples prior to consumption is a necessity to evaluate the pesticide levels in these samples. In addition, it is vital to understand the percentage of the pesticides transferred from the coffee bean to the coffee beverage to assess the factors that influence such transfer. This responsibility lies in the hands of the big industries that must run regular analysis of the coffee samples through accredited and certified labs to meet the laws of each country until we reach strict regulations about either using green pesticides or following organic farming.

In order to determine and measure pesticides in nutritional goods and the environment, many chromatographic methods have been created by utilizing GC or LC (19). These include delicate extraction techniques: liquid-liquid extraction, solid phase extraction (SPE), single-drop microextraction and solid-phase microextraction. The identification of pesticides in foods was based on the development of other extraction methods which were used to develop multi-residue methods. In the last 2 years, research has been oriented to show and prove the effect of pesticides on both human's health and psychology in addition to polluting surface water. This is to motivate a move back to organic farming proving that pesticides can kill some natural enemies that do a similar job as that of pesticides (20).

## Effect of exposure to pesticides on health and environment

The permanent presence of coffee culture for a long period of time in the field creates environmental changes which may lead to the accumulation of pests and the occurrence of diseases caused by fungi, insects, nematodes, and weeds. Harmful modifications, induced by these organisms, affect the development, production, and quality of the coffee (21). Many pesticides are implemented in order to diminish the prevalence of unfavorable organisms or plant species in coffee lands (20). Nevertheless, pesticides have toxic effects on both humans and the environment, which implies delicate control for their application and residues. The exportation of coffee represents more than 50% of the external exchange earnings in several under-developed countries. Brazil was considered, since 2008, the country that has the highest consumption rate of pesticides. The Brazilian Health Regulatory Agency (Anvisa) confirmed that Brazil's consumption of food containing pesticide residues is about 15% which may lead to damaging health effects (22). A stronger control of residues in foods, such as coffee, is crucial due to the major usage of pesticides in the agriculture field (23). One of the side effects of organo-phosphorus pesticides (OPPs) is that they block the mammalian acetylcholinesterase (AChE). This leads to the development of clinical signs related to several areas of the nervous system (24, 25). Acetylcholine levels will be automatically increased and accumulate in the muscles once cholinesterase is inhibited causing muscle disruption (26). Increased exposure to anticholinesterase compounds might seriously depress cholinesterase. In this spirit, the Tanzanian component of East Africa Pesticide Network (EAPN) evaluated the presence of clinical signs of pesticides' side effects on the Tanzanian minimal scale coffee farmers (27). It was shown that short-term spraying of OPPs might not be very harmful, but long-term spraying can be dangerous.

Due to the high consumption of coffee, both producers and consumers could be subjected to health risks due to pesticides in coffee, so attention should be drawn to the safety of coffee. A recent study proved that the exposure to pesticides increases the mortality risk for patients diagnosed with Parkinson's disease (28). Yet, more cases are to be considered to explain the reasons. Another detailed survey in Tanzania showed that acute pesticide poisoning (APP) is a health threat that is leading to an increase in the death rate for those exposed to pesticides (29). In proving the relation between the risk of hormonal exposure and risk of Parkinson's disease among postmenopausal women, the exposure to pesticides was among the factors. That is, women who were farmers or lived near farms where pesticides were used had a higher percentage of having Parkinson's disease with respect to control cases (30). Preclinical evidence proved the action of certain pesticides to increase the risk of Parkinson's. Dieldrin is an organochlorine that is widely used as an insecticide. This pesticide has been linked to neural apoptosis (31). Rotenone behaves as a neurotoxin as well. Moreover, the negative impact of pesticides is not restricted only to the farmers or consumers, but also on the future of coffee wastewater (CWW). A recent critical review showed how the use of the CWW after proper treatment could be beneficial economically and environmentally except for the minimal residues of pesticides that threaten the aquatic life even if present as trace amounts (32). This has been already confirmed in older studies showing the phytotoxicity and cytogenotoxicity in coffee wastewater (33) given that trials and attempts to remove these pesticides were initiated in 2013 and showed unsatisfactory results (34). Using coffee residues might be a way to generate extra income for coffee growers helping them offset production costs. The latter is subject to the use of pesticides. As 80 % of the population in Ethiopia depends on agriculture using uncontrolled amounts of pesticides, concerns were raised recently, and consequently, many studies in this regard have been initiated emphasizing the pesticide-related health and environmental risks. To estimate risk and develop solutions, a recent study based on an electronic database was conducted specifically on the direct use of dichlorodiphenyltrichloroethane (DDT). The data showed DDT was detected in soil, surface water, and human breast milk indicating the direct use of DDT on food crops. Moreover, this is a sign of chronic health risk to the public harming fish, bees, soil organisms, and wildlife. Hence, misuse of pesticides can lead to interruption of the entire life cycle (35). This study was a call for the necessity of raising awareness to reduce the risks resulting from pesticides' misuse.

Recent studies have been oriented to study the influence of pesticides not only on the soil, but also on the entire surrounding population. More specifically, investigations are looking at safety measures when spraying coffee beans with pesticides. For example, a recent study focused on the cytotoxic and genotoxic effects of pesticides' exposure to male farmworkers. Results showed that 87% of the farmers do not put on masks or gloves when spraying coffee crops with pesticides (36). This puts them at risk of respiratory problems. Symptoms can be clear upon either direct exposure or after a certain period of time. Despite the different levels of risk the farmers had, the problem is the same. It is true that the optimal solution is to revert to organic farming, but meanwhile it is important to take steps to control pesticide use. This could be by training farmers to handle pesticides safely. In addition to knowledge, protective clothing must be obligatory, especially in poor countries, where occupational health standards are weakly controlled. In short, with these preliminary results proving that pesticides can cause cancer, more restrictive regulations for pesticide use are needed. Serious strict control for applying these rules is required. A similar study was carried out in Thailand that came to the same conclusions about the necessity of training the farmers and providing them with protective tools for handling pesticides due to their lack of knowledge toward the pesticides' toxicity (37).

There is no doubt that the lifestyle is the key for all the health conditions including depression signs. Since Brazil is the largest coffee-producing nation, a spotlight was focused not only on the farmers' health but also on their psychological status since they have used pesticides intensively since 2008. Hence, a detailed study was conducted on the impact of pesticides on the farmers' psychology. A validated correlation between exposure to pesticides and depression was found. Mental health is another consequence to add to pesticides' use. It is true that depressive symptoms can result from many factors other than pesticides' exposure, but the latter is a factor and increases the risk as well (38). Hence, farm workers' well-being is another issue to pay attention to either by restrictive rules toward pesticides' use or by spreading awareness for rural workers so that they are followed-up with to avoid entering the cycle of depression. In an attempt to increase the yield of crops, there was a move toward using chemicals associated with a reduction of shade trees. In this regard, a study tested the relationship between distance from the forest, use of chemicals, shade cover, and the quality and quantity of butterflies that are, in turn, considered a bio indicator in coffee home gardens (39). Results showed that the use of chemicals (pesticides) affected the abundance of the butterflies negatively. On the other hand, shade trees had a positive impact on the number and the diversity of the butterflies. This is a shift toward better crop management and reducing the use of pesticides. This is, in fact, one of the reasons behind the dramatic increase of coffee production in Indonesia in the past 10 years. Organochlorine and organophosphorus pesticides were detected and quantified in the surface water next to an agricultural area planted with coffee. Water samples were subjected to microwave-assisted extraction before injecting samples to GC equipped with an electron microcapture detector to quantify endosulfan, DDT, heptachlor, methoxychlor, parathion, and chlorpyrifos (40). This shows how use of such pesticides contaminates the water and exposes peoples' health to hazardous problems and chronic diseases. Pesticides entering the water cycle are interrupting almost all the life cycles for a long time since these pesticides have long life-times.

## Detection of pesticides in coffee beans

To quantify the pesticides in foods and thus to provide good control measures, analytical techniques have always been the prerequisite to get precise results. It is widely known and agreed that gas chromatography and liquid chromatography are used (19). The choice of the technique is mainly dependent on the class of pesticides under study. That is, LC is used when the limitation of the GC is reached if the pesticides under study are polar, non-volatile, and/or thermally labile. Keys to having a validated and successful measurement lie in the choice of the spectrometric technique, and the extraction procedure using the minimum amount of chemicals, time, and cost. In addition, optimized clean-up steps are crucial depending on the food tested to avoid affecting the matrix (41–43). In our case, coffee is considered a difficult matrix to work on. This is because green coffee beans contain large amounts of sugars, organic acids, phenolic acids, important antioxidants, pigments, fatty acids, and caffeine (44, 45). Within the same chromatography, many factors can influence the validation of the method. In this part, a summary of the main key factors that solved challenges in extraction, separation, or enhancing recovery percentages is presented. The choice of the chromatography is dependent on the nature of the pesticides in question. For example, for Imidacloprid to be detected using GC, it needs to be derivatized since it is thermo-labile. If derivatization is not possible, LC should be used instead (46). In addition, the clean-up will be influenced by the choice of the chromatography. For example, to perform the GC–MS procedure, an additional clean-up was crucial. In case of LC, solvents can be run at a low flow rate after running samples so that the column is cleaned and washed by many cycles. In the case of the GC, this could not be done. As the principle of the GC, the sample evaporates and in the case of contaminants, they will block the ion source and stick on the column. Baking the column can solve a part of the problem, and cleaning the ion source is possible, but this is problematic and might alter the results of the coming samples. That is why when dealing with GC, additional caution is paid regarding the clean-up steps. For that purpose, C18 and Primary and Secondary Amines (PSA) were used as dispersive solid-phase extraction (d-SPE) sorbents. The role of C18 is to remove the lipids and esters (47) where the PSA is mainly used to eliminate more polar compounds like sugars, free fatty acids, pigments, and organic acids (48, 49). In this section, different methods and techniques are discussed and then summarized in Tables 1, 2.

**Table 1:** Summary of the detected pesticides in coffee samples, the corresponding clean-up and chromatography used.

**Publication Year**	**No of Pesticides**	**Samples**	**Detection Method**	**Clean-up**	**Reference**
2015	14	Green beans	LC-ESI-MS/MS	QuEChERS	(50)
2017	52	Coffee leaves	UHPLC-TOFMSLC/QqTOF-MS/MS	DLLME	(51)
2018	117	Ground coffee	LC-ESI-MS/MS	QuEChERS	(52)
2019	7	Roasted coffee	LC-MS/MS	LLME	(53)
2019	81	Raw beans	LC-MS/MS	SPE	(54)
2020	4	Roasted coffee	GC-ECD	NP	(55)
2022	34	Ground coffee	GC-MS	QuEChERS	(56)
2022	7	Roasted coffee	GC-MS	IL	(57)

**Table 2:** Experimental conditions for extracting coffee samples and the analytical parameters of the method.

**sample (g)**	**Extraction Solvent**	**Number of samples**	**LOQ** **(μg/kg)**	**RSD %**	**R^2^**	**LOD** **(μg/kg)**	**Recovery %**	**Reference**
10	Acetonitrile	181	0.8–9.7	< 20	>0.981	0.2–2.9	70–120	(50)
1	Acetonitrile	20	0.03–2.25	< 20	>0.99	0.01–0.25	87–94	(51)
10	Acetonitrile	—	10–50	< 20	>0.99	–	70–120	(52)
1	Acetonitrile	–	0.1	< 10	>0.99	0.02–0.05	74–99	(53)
2	Acetonitrile	–	–	–	–	–	–	(54)
3.5	Acetonitrile	–	4.45–4.77	< 5	>0.99	1.33–1.42	74–113	(55)
3	Acetonitrile:hexane (2:1)	150	3–9.3 μg/kg	< 6	>0.994	1–3.2 μg/kg	83–100	(56)
2	Dichloromethane:Acetonitrile	13	–	< 10	–	–	35–97	(57)

### QuEChERS

A method was optimized using QuEChERS (Quick, Easy, Cheap, Effective, Rugged, and Safe) for clean-up prior to analyzing 181 samples of Indonesian green coffee beans to quantify 14 pesticides using LC-ESI-MS/MS (50). This extraction method is based mainly on having a homogeneous sample in an organic solvent and then adding salts like NaCl and MgSO_4_ for dehydration followed by a clean-up achieved by means of solid phase extraction. In this part, it is important to retain the analyte under study in the extract. The triple quadrupole system coupled to this LC facilitates having two or more transition states to validate the precursor-product pairs. Samples were collected from different regions of Indonesia to show that some of them contained pesticide levels higher than the MRL. After weighing the coffee samples (10 g) in acetonitrile (10 mL), acetic acid (1 %), sodium citrate (1 g), sodium chloride (1 g), sodium hydrogen citrate (0.5 g), and magnesium sulfate (4 g) were added, and the mixture was subjected to centrifugation for 10 min. Modifying the standard QuEChERS was achieved by using graphitized carbon black (GCB). Despite the fact that GCB retains planar pesticides, it removes the pigments from the coffee bean samples (58). As for the clean-up procedure, a volume of 4 mL of the acetonitrile phase was removed and treated with PSA (150 mg), GCB (45 mg) and MgSO_4_ (855 mg) and centrifuged again. The extract was kept in the fridge overnight, and then filtered through PTFE (0.2μm) before being injected into the LC. The method developed in this study is considered a better one compared to the previous study (1). This is due to a better limit of quantification (LOQ) values compared to the maximum residual limits (MRL) required by the importing countries. In other words, this method is reliable to test tea. After validating the method and optimizing the extraction procedure using spiked samples, the method was applied to 118 samples of green coffee beans. Results proved the absence of the following pesticides: methomyl, aldicarb, diuron, and propiconazole. On the other hand, other tested pesticides were detected in different samples as presented in Table 3.

**Table 3:** The number of real samples vs. the occurrence of pesticides above LOQ with respect to different MRLs.

**Pesticide**	**Below MRLs of EU**	**Below MRLs of Japan**	**Below MRLs of US**	**Above MRLs of EU**	**Above MRLs of Japan**	**Above MRLs of US**
Carbaryl	1	1	–	3	1	–
Carbofuran	4	4	4	–	–	–
Diazinon	32	39	–	11	4	–
Diclorvos	4	1	–	–	3	–
Dimethoate Imidacloprid Malathion Methidathion Profenofos Propoxur	3 11 6 2 3 3	4 11 4 2 3 4	– 11 – – – –	1 – – – – 1	– – 2 – – –	– – – – – –

To study the impact of toxicants in coffee, 34 pesticides were detected and quantified in 150 coffee samples and risk assessment was calculated (56). Samples were extracted using QuEChERS. Coffee (3 g) was mixed with water (6mL) for 2 min, acetonitrile (12mL) and hexane (6 ml) were added, and the mixture was stirred. NaCl (1g) and MgSO_4_ (4 g) were added and centrifuged for 12 min at 3,500 rpm at −5°C. PSA were added prior to analyzing the samples using GC-MS. This was done to test the samples imported from different countries. Analysis of the samples was followed by a risk assessment calculation based on the population's consumption. Results of the hazard index indicates the high risk of health problems in addition to environmental pollution (56) if country A (anonymous indexing) was chosen. Hence, the analysis of the samples prior to consumption is an essential step to prevent and control the risk of toxicants to the consumers.

### Dispersive liquid–liquid extraction

Being one of the most consumed beverages worldwide, it is essential that coffee follows the European food safety regulations, especially when it comes to imported raw coffee since different countries have various regulations and control regarding the quality and quantity of pesticides sprayed on coffee crops. This calls attention to the necessity of analyzing pesticide residues and other contaminants that might be present in quantities exceeding the MRLs. For coffee samples to be analyzed, extraction is a must. This step is challenging due to the negative impact of caffeine present in coffee on the chromatograms obtained using the GC-MS. This is related to the large and broad peak of caffeine that blocks the analysis of the chromatogram and masks many pesticides that have retention times in the range of this wide peak (3). Additionally, caffeine can interact with the sorbent yielding a decrease in the recovery percentage. For example, in an attempt to detect the pesticides in the coffee leaves, the peaks of the 52 detected pesticides were accompanied with that of caffeine despite the use of modified QuEChERS prior to analysis using LC-MS/MS. Hence, modifications were made to enhance the results. This included testing the influence of different solid phase extractions parallel with varying the amount of C18. Despite many attempts, the caffeine peak was still a problem. As a consequence, additional extraction techniques had to be implemented with QuEchERS to have chromatograms free form caffeine peaks. In fact, QuEChERS could be coupled with dispersive liquid–liquid micro-extraction (DLLME). In this case, DLLME is used for the extraction and QuEChERS is used for the clean-up. This combination was presented in 2015 prior to quantifying organochlorine pesticides in raw coffee samples (3). Based on this, 52 pesticides in coffee leaf extracts were detected and quantified using QuEChERS combined with DLLME for extraction and clean-up steps (51). The target of the study was to analyze the coffee crops that are planted under organic conditions. For this, the aforementioned validated method was used to analyze the coffee leaf extracts from 12 traditionally grown trees and 8 organically grown trees. Results showed the absence of pesticides in all of the organic samples. Furthermore, thiametosan was detected in 6 samples taken from the traditionally grown trees.

It is worth mentioning that one disadvantage of using DLLME is the very low recovery percentage (absence sometimes) for very polar pesticides. This could be prevented by testing these pesticides using LC-MS/MS instead of GC-MS following the exact same extraction procedure with the absence of the DLLME step. This is because the caffeine peak is not an issue when using LC-MS/MS. In other words, the use of the DLLME step is dependent on both the polarity of the pesticides under study and the analytical technique used.

Studies have been carried out to show the relation between the sonication time and the recovery percentages (59). In addition, the use of both QuEChERS and dispersive liquid–liquid microextraction (DLLME) to determine pesticides in fruit samples has been reported (60). Based on these previous studies, a successful approach to determine seven pesticides present in four chemical families (carbamate, neonicotinoid, triazole, and organophosphate) in roasted coffee (*Coffea Arabica*) was demonstrated combining the ultrasonic solvent extraction method with DLLME as the cleaning step prior to analysis using ultra-performance LC-tandem MS (UPLC-MS/MS) technique (53). Coffee samples were grinded and stored. Then, samples (1g) were spiked with standard solution (500 μL), with specific concentrations (0.1, 0.2, 0.3, and 0.5 μg/kg). For each fortification level, three replicate analyses were performed. Several parameters regarding the procedure were assessed such as solvent type and quantity and sonication time. Optimized conditions could be summarized as testing roasted coffee (1 g), in acetonitrile (5 mL) having an optimal sonication period of 15 min, and dichloromethane (1 mL) as the extraction solvent in the clean-up step. The average recoveries ranged from 74.3 to 99.9%, with RSDs ranging from 0.7 to 10.2%. These results prove that the combination of ultrasonic solvent extraction parallel with the optimal sonication time and DLLME (cleaning step) demonstrated accurate and precise measurement of the residues. The LOD values had a range between 0.02 and 0.05 μg/kg, where LOQ had a value of 0.1 μg/kg for all the residues. In order to evaluate the repeatability of the procedure, nine consecutive analyses of 0.1 μg/kg pesticide standard solutions were performed, and the RSDs ranged from 1.1 to 10.2%. The procedure demonstrated a good linearity for all the pesticides by observing correlation coefficients >0.99. The results of analyzing real coffee samples manufactured in Brazil showed that they are pesticide-free. Despite the absence of pesticides, this study yielded a method that has better accuracy, precision, LOD, LOQ, and RSD values. Hence, it is selective for multiresidue analysis of pesticides when compared to analysis of the same pesticides done by Dias et al. (2) and Yang et al. (1). In addition, LOQs were compatible with respect to the limit values established by Brazilian legislation and the Codex Alimentarius. Furthermore, it could be described as an economical procedure since only a small sample size was needed for the performance of the procedure providing significant savings at the level of solvent, consumables, and analysis time.

### Dilution

Therefore, a main focus of this study was to optimize the QuEChERS procedure, omitting the least needed steps, and highlighting the most important steps for identification of several pesticides present in the complex coffee matrix. It was necessary to optimize the QuEChERS procedure by performing tests with acetonitrile acidified with acetic acid or formic acid, with or without a buffer and with or without clean-up of the extracts before the LC-ESI-MS/MS step. Afterwards, the clean-up approach consisted of the evaluation of seven d-SPE sorbents and their several mixtures. A validation method was then conducted in order to obtain an efficient performance for the extraction and chromatographic procedures. The removal of the buffer and clean-up steps from the procedure showed an efficient extraction, specifically in decreasing the waste. The samples were spiked with pesticides at 3 concentration levels with 6 replicates at each concentration followed by the different steps of extraction and the two extracts underwent dilution two-fold with methanol containing propoxur as I.I.S for the purpose of technique improvement of the early eluting polar analytes. Afterwards, the LC-ESIMS/ MS procedures were used for pesticides analysis. For the optimization of the QuEChERS approach, using as an extraction solvent, acetonitrile acidified with 1% acetic acid, without performing a clean-up step, proved the validation of the procedure. In addition, the two-fold dilution of samples prior to testing improved LC results. That is, upon decreasing the concentration by dilution of the extracted sample, resolution of peaks was better. In addition, this guarantees that the concentration of the sample fits in the calibration curves set, and results are enhanced. In summary, 117 out of 131 pesticides (89%) showed good results (recovery within the range of 70–120% and RSD < 20%) due to diluting samples prior to testing (52). It is worth mentioning that this study was targeted to show the influence of diluting the samples parallel with reducing the clean-up steps to get faster, greener, and more economical methods to analyze pesticides in coffee samples. This has been applied to spiked samples proving the reliability of the method to test real coffee samples when needed.

### Solid phase extraction

Carbon spheres have functional groups like carboxyl or hydroxyl that make the adsorption of metal precursors easier (61, 62). Oxides are non-toxic, and stable chemically and thermally. Additionally, they are of low cost. Due to their porous structures, they have many uses in the technology of separation (63). The functional groups on such composites provide selectivity in the interaction with the target molecules (64). In this regard, it could be used for the extraction step when analyzing pesticides. A recent study in 2020 illustrates the synthesis and implementation of a magnetic amino-functionalized hollow silica-titania microsphere (SiO2-TiO2- NH2@Fe3O4), identified as the sorbent for magnetic dispersive micro-solid phase extraction (MD-μ-SPE) of four pesticides in coffee bean samples (55). The structure of the sorbent was characterized by Fourier infrared spectroscopy, field emission scanning electron microscopy, transition electron microscopy, energy-dispersive X-ray spectroscopy, and vibrating sample magnetometer procedures. This method is characterized by the use of nanosorbents doped with magnetic nanoparticles (NPs) in a batch mode in order for the analytes to be rapidly extracted. Afterwards, the separation of the sorbent from the sample solution was performed by a handheld magnet. Therefore, centrifugation or filtration phases were not conducted. Hence, selective extraction occurred due to the possible alteration of the surface functionality of the sorbent. Afterwards, the residues were analyzed using the gas chromatography-electron capture detection (GC-ECD) method. The magnetic solid phase extraction was optimized by considering the following parameters: desorption conditions such as desorption solvent and time, sorbent quantity, extraction time, and pH of the sample solution and the salt concentration. The highest recoveries were obtained by using ethyl acetate (0.6 mL), vortex time (4 min), sorbent (25 mg), and extraction time of 15 min at pH = 6. The extraction time is important since it is related to the adsorption equilibrium where sufficient time of contact between the sorbent and the analytes is needed (65). Another important factor that plays a key role is the pH control. Hydrolysis of the target analytes was observed at low pH (strongly acidic) and high pH (strongly basic) (66, 67). The method presented in this study showed high performance and applicability in identifying pesticides in green and roasted coffee bean samples with good recoveries between 74 and 113% for the spiked samples. Therefore, the used sorbent can be implicated for the (MD-μ-SPE) of pesticides in complex matrices, such as a plant-derived food matrix by eliminating the clean-up phase including the centrifugation or filtration. This reduces both time and cost. In addition, when compared to different methods to detect the same pesticides reported in literature, this method is more efficient, has better recovery, precision, and higher sensitivity when it comes to the LOD and LOQ (2, 51, 52, 54).

### Ionization mode

The characteristics and the design of the origin of ionization impact the execution of a bioanalytical technique such as LC-MS/MS when analyzing pesticides. UniSpray (US) ionization, also called impactor ionization, is an atmospheric ionization procedure using high-velocity spray for the ionization of analytes (68). However, it provides additional droplet break-up, desolvation, and enhanced performance when compared to electrospray ionization (ESI). Analyzing various samples is critically affected by the matrix effect due to its components that can enhance or suppress the ionization step in the mass spectrometer. A recent research study evaluated the effect of US compared to ESI, used in the multi-residue analysis of pesticides, on the same LC-MS/MS. The assessment of both interfaces was demonstrated by performing an analysis on three different matrices (coffee, apple, and water), in order to conclude if the multi-residue analysis of 81 pesticides by QuEChERS (acetonitrile extraction; PSA and C18 clean-up) and LC-MS/MS analysis, have an improvement possibility (54). The parameters reflecting the analytical execution such as signal intensity, signal-to-noise (S/N) ratio, linearity, accuracy, precision (%RSD), relative abundance (ion ratio), extraction recovery, sensitivity (LOD and LOQ), and ME, as well as recovery efficiency (RE) and PE were assessed and compared.

When only considering the matrix effect regardless of the chemical class, a significant gain in signal intensity (22- to 32-fold in peak area, six to seven-fold in peak height) was observed with US. A rise of threefold to fourfold in signal-to-noise ratio was also observed. Adding to it, the linearity and precision were comparable between UniSpray and ESI. The total LOD and LOQ did not show any significant ranging between the two ionization interfaces. Several components showed a gain in sensitivity with US. ESI had higher signal suppression than US which led most of the ME values to be within the satisfying variation. The matrix effect was 3 to 4 times higher, but more satisfying compared to ESI. No difference was observed between ESI and US regarding the recovery efficiency in different matrices. Thus, the amelioration of the process efficiency reached 3 to 4 times higher progress with US compared to ESI (54).

### Ionic liquids

The extraction phase could use ionic liquid (IL) as well. Ionic liquids (ILs) are organic salts made up of non-toxic ions that are liquid at room temperature. ILs are named as a designer solvent due to their interesting physical properties that play a role in various applications such as separation. In addition of being friendly to the environment, they enhance sensitivity, selectivity, and accuracy (69). Furthermore, their properties can be tunable depending on the application. They are known to have a low melting point, elevated thermal stability, low vapor pressure, high extractability, and air-moisture stability (70). In this attempt, IL was synthesized, supported on silica, and then functionalized with graphene oxide to serve as sorbents used for microextraction in the aim of detecting OCPs (diazinon, heptachlor, aldrin, endrin, dieldrin, endosulfan, and methoxychlor) in coffee samples (57). This created phase was compared to other sorbents where some are functionalized with graphene as well, but it gave the best results due to its selectivity and adsorption capacity that were evaluated by the GC. In turn, this method was later adapted for detecting pesticides in 13 coffee samples. Results showed that all pesticides are not detected using this methods' parameters except for methoxychlor. The latter was present in five of these samples with concentrations ranging between 29.5 and 180.02 μg/L.

#### Birds as bio indicators

Birds are considered as good bioindicators since they are sensitive to toxicants, observable, and live in various trophic areas. To understand the relationship between diseases and health, it is vital to analyze the interactions between the organisms and the environment (71). Human activities implemented the use of chemical compounds that have had impacts on many animal species. Add to this, the environmental changes that have been monitored chemically and physically. Birds have been playing as an essential role as bioindicators since 1916 when canaries were used in mines (72). Later in 1965, hawks and seabirds were used as indicators for pesticide accumulation (73). The latest method to detect contamination specifically due to pesticides is by the aid of birds as bio indicators (74).The cytologic technique is used in this assessment following the results of the DNA. In particular, a study conducted tests to measure the efficiency of the blue-black grassquit in responding to *in-situ* pesticides' contamination in various-sized coffee farms in Brazil. The erythrocytes of the birds were tested by the micronuclei test. By comparing the results to the results of tests using established techniques to test for contamination by pesticides, the method proved to be sensitive and efficient enough to be used on coffee farms. The evolution of technology resulted in the transfer from the laboratory to practical needs matching operational simplicity. For instance, fabrication of large-scale surface-enhanced Raman scattering (SERS) substrates *via* a simple strategy was reported in 2018 (75). It is worth mentioning that neither complicated instruments nor toxic chemicals were needed. First, Ag nanomaterials (NPs) were synthesized. After having a drop of the Ag NPs dry on a horizontal surface, a layered structure will form, and by evaporation clingy resistance loads the capillary flow. In this way, the coffee-ring effect is removed and as a result AG NP deposits. In turn, these deposits function as active layers to detect pesticides in coffee. This method is economical, efficient, and could be applicable on a large scale with high precision and with no extraction or clean-up steps.

## Transfer of pesticides before infusion preparation

The testing of pesticides is affected by the processing methods a coffee bean passes through. Following a precise protocol can influence the quantity and quality of pesticides present in the coffee beans and hence the influence of these pesticides on human health upon consumption. A study has been conducted to determine the dinotefuran (an insecticide of neonicotinoid class) and its metabolites during the different steps—washing, roasting, and brewing the beans; preparing the infusion-using LC-MS (76). The aim of the study was to prove the necessity of the follow-up of transfer of pesticides before having the infusion ready to be consumed taking into consideration the half-life of the pesticide of interest. Neonicotinoid are considered the best insecticides which work on insect nicotinic acetylcholine receptors and are by far the most successful insecticides (77, 78). Dinotefuran (RS)-1-methyl-2-nitro-3-(tetrahydro-3-furylmethyl) guanidine is a recent furanicotinyl and a part of the third generation of neonicotinoid insecticides (79). Dinotefuran has maximum residue limits ranging between 0.05 and 25mg·kg^−1^ in several consumable goods. The reasonable daily intakes range between 0 and 0.2 mg kg^−1^ body weight (bw) and the acute reference doses are around 1 mg kg^−1^ bw. Their contamination of raw and processed (washing, roasting, and brewing) coffee beans has been rarely mentioned in the literature. However, it is also crucial to investigate the impact of these processes on pesticide residues and their metabolites in coffee beans. Therefore, in order to detect dinotefuran in coffee beans, an advanced sensitive and specific technique using Florisil solid phase extraction (SPE) cartridges combined with liquid chromatography-tandem mass spectrometry (LC-MS/MS) was established. A dissipation assessment of dinotefuran in coffee beans was conducted in Yunnan province, China, following supervised field trials with specialized agricultural monitoring. Then, washing, roasting, and brewing processes were conducted. An analysis of wash water and coffee sludge was then performed. A matrix-matched calibration standard for the quantification of residues in coffee-bean samples by the utilization of LC-MS/MS analyses was done. The Kruskal-Wallis test was performed for the determination of percent reductions of pesticide after washing, roasting, and brewing. Matrix-matched calibration curves were used and matrix effects were calculated following equations as mentioned in literature (80). Dinotefuran had a recovery in coffee beans (before and after processing) and water ranging between 73.5 and 106.3% with relative SDs < 10% and regression coefficients (*r*^2^) > 0.997. Dinotefuran had limits of detection and quantification of 0.003 and 0.01 mg kg^−1^. The analyses of the coffee-bean samples during 2015 and 2016 demonstrated initial deposits of dinotefuran between 2.59 and 2.86 mg kg^−1^ and a mean half-life of 40.8 day. After being washed for 5 min under tap water where temperature ranges between 25 and 30°C, raw and processed coffee beans observed a decrease in concentrations of dinotefuran, by 44.4–86.7%. It is worth mentioning at this stage that the washing step is related to many factors, such as the temperature and the solubility of the pesticide of interest in addition to the age of the residue (81). The roasting process that took place at 230–240°C for 12–14 min diminished the concentrations of dinotefuran by 62.2–100% with the highest reductions between 1 and 14 days. Placed in boiling water for 12 min, Dinotefuran was not present in sludge at 28 days after brewing, while dinotefuran was diminished by 92.9–100% in the upper liquid layers. The brewing was the most successful step for removing dinotefuran residues from coffee beans compared to the washing and roasting steps. The Kruskal-Wallis test demonstrated significant differences in the consistency of the residues in coffee beans between the several steps of processing (*P* ≤ 0.05). The washing step reduced the concentration of pesticides less than the roasting (*P* = 0.0001) and brewing (*P* = 0.002) steps. Dinotefuran was detectable in coffee sludge and wash water at 1–56 days following crop treatments. Therefore, it is necessary to avoid consuming coffee sludge when drinking brewed coffee as suggested by Mekonin et al. (82). To conclude, the recommended maximum exposures to pesticide residues normally do not cause harmful effects to the overall population during the collection and processing of coffee between 28 and 35 days following pesticide treatment where dinotefuran was in the allowed recommendation. However, coffee-bean wash water and coffee sludge are proven to cause contamination.

## Mitigation techniques

In attempts to avoid and limit using pesticides for coffee, alternatives were suggested such as using alcohol traps targeting the reduction of insects' generations in Vietnam. This was to limit the risks to farmers and consumers due to using pesticides. Studies are now looking to find solutions other than using pesticides to protect the coffee crops from being attacked by insects. A recent study showed that using alcohol traps at specific times of the year was effective to kill the female coffee berry borer (CBB). In addition, the use of Beauveria bassiana was more effective when compared to using insecticides to reduce the level of CBB (83).

Many attempts and steps are currently being taken in different parts in the world to spread awareness about the quality and quantity of pesticide use. In addition, tips are provided to obtain optimal results. An example could be what was recently reported in Indonesia regarding training the farmers about spacing of coffee seeds, spacing and types of protective trees, and pruning coffee and protective plants to get the best results at the level of coffee beans parallel with the minimum use of pesticides (84).

The solution is not avoid drinking coffee since even non-consumers are exposed to pesticides used for coffee beans. It is because these pesticides will pollute underground water and hence be a pollutant that will disrupt all life cycles. For instance, the aquatic cycle in Brazil is in danger. A study was conducted in regard to pesticides sprayed on coffee plantations evaluating the percentage of the pesticides that go into the ground. It was shown that by using pesticides, there is a chance of 45 and 24% for these pesticides to contaminate the surface water and the ground water, respectively. More specifically, ametryn, cyproconazole, diuron, epoxiconazole, flutriafol, triadimenol and triazophos pose a risk to contaminate both the ground water and the surface water (85). This is a factor to be added to the regulations where, if the use of pesticides is a necessity, then the location must be away from water sources in an attempt to reduce the risk of such contamination. A similar study was also conducted in Rwanda (86). In particular, malathion was detected in surface water at concentrations above the threshold posing a risk for arthropods. Moreover, the surface water was polluted by malaxyl and carbendazim. This could be related to the fact that they are the most used pesticides by farmers there. Respiratory problems could be a consequence of exposure to pesticides. It is true that many factors can lead to respiratory issues, but this has been proven in Rio De Janeiro upon performing cholinesterase tests for family farmers in the crop-season and spirometry in both crop- and off-season (87). In fact, they had 40, 31, and 24% for coughs, nasal allergies, and chest tightness, respectively. It is true that exposure to pesticides is not the only reason for such diseases, but there is no doubt that it is a factor that increases the risk and could be a reason of chronic diseases.

An alternative solution could be to start moving toward organic cultivation. This is based on recent studies that are based on calculations and comparisons. An example is a recent study that was published in 2022 mentioning the results of a three-year follow-up for coffee corps evaluating the effect of the cultivation system of coffee against the attack of the coffee berry borer in Indonesia (88). For 3 years, coffee plants were planted organically and conventionally. Surprising results showed that the attack of the CBB was similar on both the organic and the conventional coffee plants. This tells that the use of pesticides did not have a significant influence. Add to this the cost of buying pesticides, the negative impact on the environment, water surfaces, and human health and psychology. Results were interpreted, and it was explained that the CBBs were attacked by natural enemies under organic conditions; this explains the similar levels of attack by CBBs in the presence of pesticides. This can be related also to the possibility that the pesticides are limiting the population of natural enemies.

## Conclusion

In conclusion, an overview of the pesticides in coffee was presented. Research has been conducted to quantify pesticides in coffee beans, coffee leaves, and coffee infusions. Many factors have been optimized to provide valid methods that could be adapted to test coffee samples. Pesticides must be used on all crops including coffee. It is not a solution to avoid coffee. This study shows the importance of controlling the type and the quantity of pesticides used. Then using the methods validated, calculations of transfer of pesticides from the coffee bean to the body of the consumer must be done. This permits the safe daily consumption based on the consumer's body weight. In addition, proper training and qualifications are needed to handle pesticides. This can be initiated by farmers to reduce the negative impacts on public health and the environment. New strategies can be applied to reduce pesticide residues in coffee, and in turn reduce their impact on human health and the environment. Integrated management is needed. For example, producers must adapt phytosanitary measurements parallel with studying the type of soil. Plant diseases should be controlled with rational use of pesticides that are environmentally safe, less toxic, and avoid the choice of resistance strains. On the other hand, recent studies have called for organic farming or providing natural solutions rather than the use of pesticides.

## Author contributions

AM analyzed articles in the literature and co-wrote the manuscript. RK co-wrote the manuscript. HH conceptualized the project and co-wrote the manuscript. All authors contributed to the article and approved the submitted version.

## Conflict of interest

The authors declare that the research was conducted in the absence of any commercial or financial relationships that could be construed as a potential conflict of interest.

## Publisher's note

All claims expressed in this article are solely those of the authors and do not necessarily represent those of their affiliated organizations, or those of the publisher, the editors and the reviewers. Any product that may be evaluated in this article, or claim that may be made by its manufacturer, is not guaranteed or endorsed by the publisher.
